# Anti-Biofilm Activity of Cocultimycin A against *Candida albicans*

**DOI:** 10.3390/ijms242317026

**Published:** 2023-12-01

**Authors:** Xiaohong Zhu, Anqi Wang, Yifan Zheng, Dan Li, Yuanjuan Wei, Maoluo Gan, Yan Li, Shuyi Si

**Affiliations:** Beijing Key Laboratory of Antimicrobial Agents, Institute of Medicinal Biotechnology, Chinese Academy of Medical Sciences and Peking Union Medical College, Beijing 100050, China; zhuxiaohong@imb.pumc.edu.cn (X.Z.); wanganqi@imb.pumc.edu.cn (A.W.); zhengyifan@imb.pumc.edu.cn (Y.Z.); lidan2000125@163.com (D.L.); weiyuanjuan@imb.pumc.edu.cn (Y.W.); sishuyi@imb.pumc.edu.cn (S.S.)

**Keywords:** cocultimycin A, *C. albicans*, biofilms, adhesion, hypha

## Abstract

*Candida albicans* (*C. albicans*), the most common fungal pathogen, has the ability to form a biofilm, leading to enhanced virulence and antibiotic resistance. Cocultimycin A, a novel antifungal antibiotic isolated from the co-culture of two marine fungi, exhibited a potent inhibitory effect on planktonic *C. albicans* cells. This study aimed to evaluate the anti-biofilm activity of cocultimycin A against *C. albicans* and explore its underlying mechanism. Crystal violet staining showed that cocultimycin A remarkably inhibited biofilm formation in a dose-dependent manner and disrupted mature biofilms at higher concentrations. However, the metabolic activity of mature biofilms treated with lower concentrations of cocultimycin A significantly decreased when using the XTT reduction method. Cocultimycin A could inhibit yeast-to-hypha transition and mycelium formation of *C. albicans* colonies, which was observed through the use of a light microscope. Scanning electron microscopy revealed that biofilms treated with cocultimycin A were disrupted, yeast cells increased, and hypha cells decreased and significantly shortened. The adhesive ability of *C. albicans* cells treated with cocultimycin A to the medium and HOEC cells significantly decreased. Through the use of a qRT-PCR assay, the expression of multiple genes related to adhesion, hyphal formation and cell membrane changes in relation to biofilm cells treated with cocultimycin A. All these results suggested that cocultimycin A may be considered a potential novel molecule for treating and preventing biofilm-related *C. albicans* infections.

## 1. Introduction

Fungal infections, especially of the deep-fungal type, are among the leading causes of death in seriously ill or immunocompromised individuals, such as cancer patients, transplant recipients, and those infected with HIV. *C. albicans* is an opportunistic species that is chiefly responsible for fungal infections [1]. In 2022, the World Health Organization (WHO) reported fungi in the list of priority pathogens for the first time, in which *C. albicans* was listed in the severe-grade group of 19 fungi that pose the greatest threat to public health [2].

*C. albicans*, usually a harmless commensal in healthy hosts in the yeast phase, colonizes the gastrointestinal tract, reproductive tract, mouth, and skin. When the body is immunocompromised or exposed to local environmental changes, *C. albicans* can overgrow or even induce invasive infections, which are mainly characterized by biofilm formation [3,4,5,6]. Biofilm formation is initiated by the adherence of yeast cells to the substrate (adherence or seeding), which then proliferate rapidly (initiation stage), eventually producing early-stage filamentation (hyphae and/or pseudohyphae) [7,8,9]. This is followed by an accumulation of extracellular matrix (ECM), which incorporates the network of polymorphic cells and provides a structured appearance to the biofilm (maturation stage) [10,11]. The final stage of biofilm formation is the dispersal stage, in which round yeast cells are released to seed new substrates [7,8,9]. During the formation of *C. albicans* biofilms, the adherence of yeast cells to the tissue facilitates penetration and host immune evasion, and hyphae can secrete various virulence factors that damage the host tissue. The hyphal form is resistant to phagocytosis and increases the adherence of *Candida* cells to the host epithelial cells, and the transition from yeast to hyphae represents a key virulence factor of *C. albicans* [12]. Meanwhile, biofilm formation is an important factor contributing to resistance against antifungal drugs [13,14]. Compared with planktonic cells, highly structured biofilms are composed of yeast cells, pseudohyphal cells, and hyphal cells (chains of cylindrical cells), which are interspersed with a polymeric ECM that covers and protects the cells, exhibiting resistance to antifungal drugs [9,15,16,17]. Moreover, biofilms occur not only in the mucosa or endothelium involved in the development of common candidiasis, such as vaginal and oral candidiasis, but also in medical devices, such as vascular and urinary catheters and dentures [18]. Furthermore, cells in mature biofilms are free to disperse and colonize other areas, which can form new foci [19]. Therefore, the key role of biofilms in this variability in pathogenicity and drug resistance of *C. albicans* increases the challenge of finding an effective solution to tackle the threat of *Candida* biofilms. Thus, the evaluation of antifungal drugs that inhibit biofilms besides planktonic cells is an important area of research.

Antifungal drugs derived from natural products play an important role in the treatment of fungal diseases in clinical practice. Currently, among the three major classes of antifungal drugs used in clinical practice, two classes (polyenes and echicandins) are natural products or derived from natural products. In addition, a number of first-in-class compounds derived from natural products being evaluated in clinical and preclinical trials showed promising antifungal potential [19,20,21]. As part of our ongoing search for new antifungal agents from marine microbes, we utilized the co-culturing strategy to activate the expression of cryptic secondary metabolic biosynthetic genes [22,23], resulting in the discovery of a novel chemical class of antifungal compounds, cocultimycins A–E, from *Aspergillus versicolor* IMB17–055 co-cultured with *A. chevalieri* [24,25]. During the same time, another research group from Australia investigated the Australian fungus *A. burnettii* and reported the isolation of burnettramic acid A, which was proposed to possess the same structural skeleton as cocultimycin A but with the hydroxy groups and the double bond substituted at different positions [26]. Through a comprehensive analysis of spectroscopic data and chemical degradation, we revealed that burnettramic acid A and cocultimycin A were likely to be the same compound and revised the proposed structure of burnettramic acid A [27]. Cocultimycins A–D (also named burnettramic acids A and C–E, respectively) showed significant antifungal activity in vitro against planktonic *C. albicans*, *Cryptococcus*, yeast, and mold with the minimum inhibition concentrations (MICs) in the range of 0.5–4 μg/mL [27]. Herein, we further reported the inhibitory activity and mode of action of the major product cocultimycin A against *C. albicans* biofilm formation and mature biofilms.

## 2. Results

### 2.1. Cocultimycin A Inhibited Biofilm Formation and Disrupted Mature Biofilms of C. albicans

**Figure 1:**
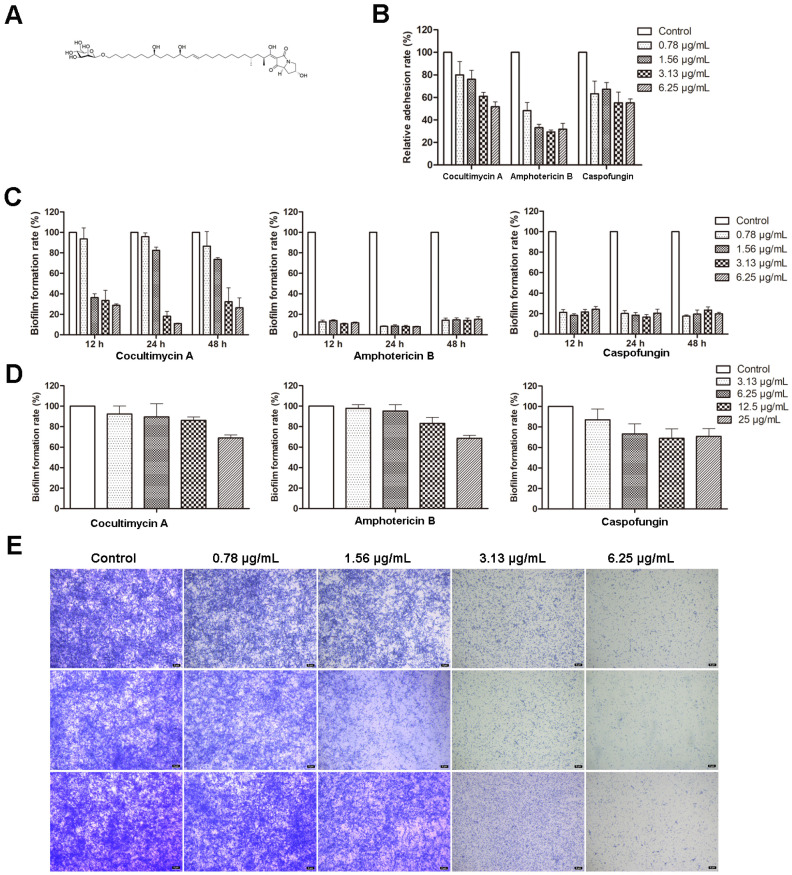
Effect of cocultimycin A against *C. albicans* biofilm. *C. albicans* cells in polystyrene 96-well cell plate were treated with cocultimycin A or DMSO (0.1%) at different times. The crystal violet staining method was used to detect *C. albicans* biofilms’ total biomass. The results were means ± SEM for three independent experiments. (**A**): Structure of cocultimycin A. (**B**): Cocultimycin A inhibited the adhesion of *C. albicans cells* to polystyrene plate. (**C**): Cocultimycin A inhibited the formation of *C. albicans* biofilm. (**D**): Inhibitory activity of cocultimycin A on muture biofilm. (**E**): Biofilm inhibitory activity of cocultimycin A with crystalline violet staining. The biofilm treated with cocultimycin A or DMSO (0.1%) for 12 h, 24 h and 48 h were stained with 0.1% crystal violet for 20 min and observed using a light microscope at a magnification of 10×, and scale bar is 5 μm.

The process of biofilm formation by *C. albicans* involves several steps, namely, early (0–11 h), intermediate (12–30 h), mature (31–72 h), and possibly a fourth dispersal stage that involves the production and release of less-adherent cells from mature biofilms. In this study, the antibiofilm activity of cocultimycin A (Figure 1A) against *C. albicans* at different stage was determined using the crystal violet quantification method. As shown in Figure 1B, treated with cocultimycin A for 2 h, the number of cells adhering to the 96-well plate was significantly reduced in a dose-dependent manner. The relative adhesion rate, when treated with 0.78 µg/mL cocultimycin A, decreased to 80.08%, and at 6.25 µg/mL, it was only 51.73%, which indicated that the adhesion ability of *C. albicans* cells was significantly inhibited by cocultimycin A. The rate of biofilm formation of groups treated with cocultimycin A also decreased in a dose-dependent manner at 12, 24, and 48 h, while that of groups treated with 6.25 µg/mL cocultimycin A was only 28.80% (12 h), 10.95% (24 h), and 26.45% (48 h), respectively (Figure 1C). For mature biofilms, Cocultimycin A only exhibits a relatively weak scavenging ability at high concentrations, and the rates of biofilm formation of groups treated with 25 µg/mL cocultimycin A decreased to 68.94% (Figure 1D).

The biofilm inhibitory activities of two clinically proven antifungal drugs for the treatment of invasive fungal infections, amphotericin B and caspofungin, were also evaluated in this study as positive controls. As shown in Figure 1B–D, amphotericin B showed significantly better inhibitory activity against biofilm formation compared with that of cocultimycin A. However, caspofungin at lower concentrations (0.78 µg/mL) showed superior inhibitory activity against biofilm formation than that of cocultimycin A, whereas, at higher concentrations (1.56, 3.13 and 6.25 µg/mL), it showed similar inhibitory activities to cocultimycin A. For mature biofilms, as with cocultimycin A, amphotericin B and caspofungin also showed very low disruptive activity, even at 25 µg/mL.

The effect of cocultimycin A on biofilms was also observed under a microscope using crystal violet staining. As shown in Figure 1E, samples treated with DMSO were stained dark blue, with a high density of biofilm and slender hyphae crisscrossing to form a dense network structure. In contrast, the staining and reticulation density in groups treated with cocultimycin A significantly decreased dose-dependently.

All these results suggest that cocultimycin A inhibited both biofilm formation and showed relatively weak disrupted activity on mature biofilms at high concentrations against *C. albicans*.

### 2.2. Effects of Cocultimycin A on Metabolic Activity of Mature Biofilm

**Figure 2:**
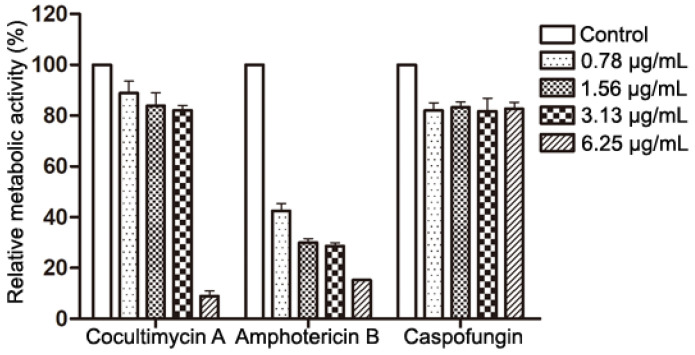
Effects of Cocultimycin A on the metabolic activity of mature biofilms. *C. albicans* cells were seeded in a 96-well plate and incubated at 37 °C for 24 h and treated with cocultimycin A for another 24 h. The XTT reduction method was used to evaluated metabolic activity of biofilm. The results were means ± SEM for three independent experiments.

Because crystal violet can stain both dead and living cells, this staining method only displays the biomass of the biofilm but not the metabolic activity of the cells in the biofilm. Therefore, the XTT reduction method was used to further evaluate the effect of cocultimycin A on the metabolic activity of the mature biofilm. As a substrate for mitochondrial dehydrogenase, XTT can be reduced to water-soluble formazan by enzymes in the cytoplasm of the respiratory chain, which can indicate the amount and metabolic activity of living cells. Compared with the control, at 6.25 μg/mL, cocultimycin A only reduced the biomass biofilm to 89.55%, but the relative metabolic activity decreased to 8.96% (Figure 2).

### 2.3. Cocultimycin A Decreased the Hydrophobicity of C. albicans and HOEC Cell Adhesion

**Figure 3:**
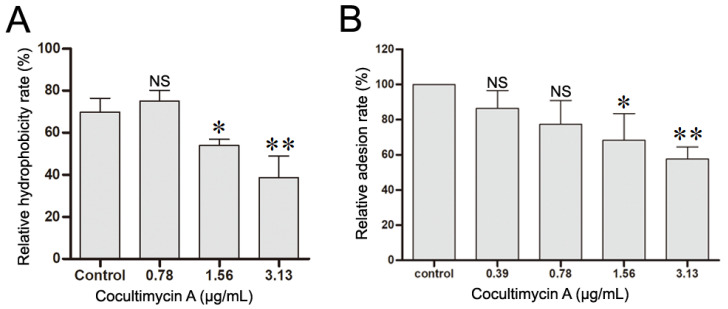
Cocultimycin A down-regulated the hydrophobicity and adhesion of *C. albicans* cells to HEOC. (**A**): Cocultimycin A reduced the hydrophobicity of *C. albicans* biofilm. The working suspension in 6-well cell culture plates was treated with cocultimycin A (0.78–3.13 μg/mL) or DMSO (0.1%) for 24 h. The biofilm cells were resuspended in PBS buffer and CSH was determined by using the water–hydrocarbon two-phase method using methylbenzene as the organic phase. The results were means ± SEM for three independent experiments. NS, no significant difference; *, *p* < 0.05; **, *p* < 0.01. (**B**): Cocultimycin A inhibited the adhesion of *C. albicans* cells to HOEC cells. *C. albicans* cells in YEPD broth (1 × 10^5^ celld/mL) were treated with cocultimycin A (0.39–3.13 μg/mL) or DMSO (0.1%) for 12 h at 30 °C, and then were collected by centrifuging (5000 rpm, 5 min). *C. albicans* cells were then resuspended with RPMI-1640 to 1 × 10^7^ cells/mL (0.1 mL) mixed with HOEC cells (0.1 mL) and incubated at 37 °C for 1 h. The number of *C. albicans* cells adhering to randomly selected 100 HOEC cells was calculated. The results were means ± SEM for three independent experiments. NS, no significant difference; *, *p* < 0.05; **, *p* < 0.01.

CSH is an important virulence factor of *C. albicans* and plays a vital role in the adhesion of *C. albicans* on media and biofilm formation. The higher the value of CSH, the greater the ability of *C. albicans* adhesion and biofilm formation [28,29,30,31,32]. Here, we investigated the influence of cocultimycin A on the CSH of *C. albicans* and observed that the hydrophobicity of *C. albicans* biofilms was down-regulated by cocultimycin A dose-dependently. The CSH value of the DMSO-treated sample was 69.86%, whereas, for biofilms treated with cocultimycin A, it significantly decreased to 54.07% (1.56 µg/mL) and 38.64% (3.13 µg/mL) (Figure 3A).

Moreover, the effect of cocultimycin A on the adhesion activity of *C. albicans* was further evaluated using HOEC cells, which are the hosts of *C. albicans* in oral infections. As shown in Figure 3B, the percentage of *C. albicans* cells adhering to HOEC cells was significantly decreased following treatment with cocultimycin A compared with the control group in a dose-dependent manner, and the relative adhesion rate decreased to 77.37% at 0.78 µg/mL, 68.33% at 1.56 µg/mL, and 57.58% at 3.13 µg/mL.

### 2.4. Cocultimycin A Weakens the Filamentous Development of C. albicans

**Figure 4:**
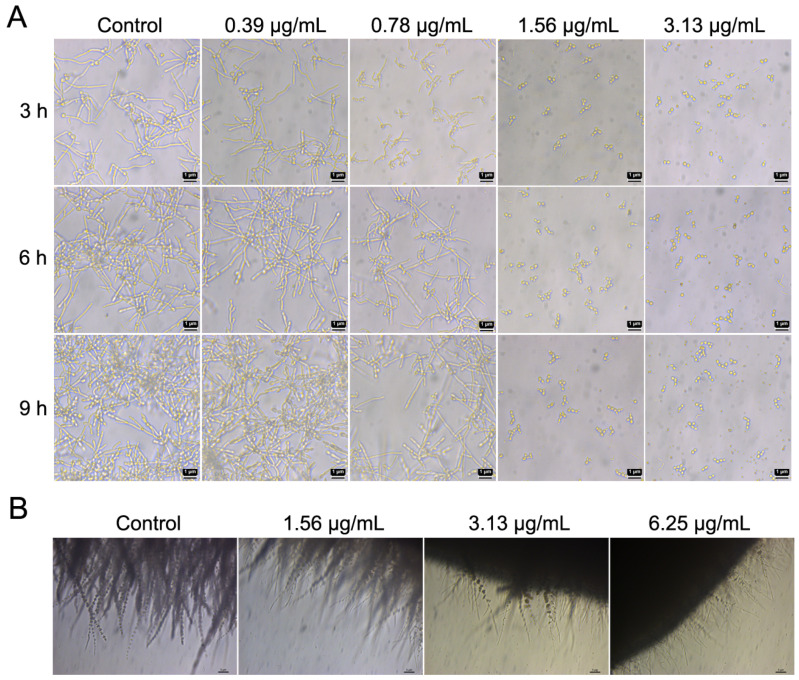
Cocultimycin A inhibited the formation of hyphae in biofilm of *C. albicans*. (**A**): Cocultimycin A inhibited the yeast-to-hypha phase transition of *C. albicans* cells. *C. albicans* cells were treated with cocultimycin A or DMSO (0.1%), and then the morphology of cells at 3 h, 6 h and 9 h were observed via light microscope at a magnification of 40×, and the bar is 1 μm. (**B**): Cocultimycin A inhibited the mycelial formation of *C. albicans* colonies. The working suspension drop (0.5 μL) was transferred to the YEPD solid plate containing cocultimycin A (1.56–6.25 μg/mL) or DMSO (0.1%) and incubated at 37 °C for 7 days. The hyphae of colony were observed with light microscope at a magnification of 10×, scale bar is 5 μm.

Besides adhesion, morphological transition is also an important virulence factor contributing to *C. albicans* biofilm formation, and the formation of hyphae is a main characteristic of *C. albicans* biofilms [33,34]. After the adherence of *C. albicans* for 2 h, cocultimycin A was added to observe its effect on its morphology under an optical microscope. As shown in Figure 4A, the yeast-to-hypha phase transition was inhibited by cocultimycin A. Cells treated with DMSO formed long hyphae at 3 h and exhibited mycelia-wrapped microcolonies at 9 h, whereas, among groups treated with cocultimycin A, treatment with 0.78 µg/mL cocultimycin A resulted in the formation of only germ tubes at 3 h and short hyphae (6 h and 9 h), while 1.56 µg/mL and 3.13 µg/mL cocultimycin A could completely restrict the cells to the yeast phase.

Colony hyphal formation also further confirmed the inhibitory activity of cocultimycin A on *C. albicans* hyphal formation. On a solid medium with DMSO, colonies formed by *C. albicans* showed abundant filamentous morphology under the microscope. On media containing 3.13 µg/mL and 6.25 µg/mL cocultimycin A, the quantity and lengths of the filaments were significantly decreased (Figure 4B).

**Figure 5:**
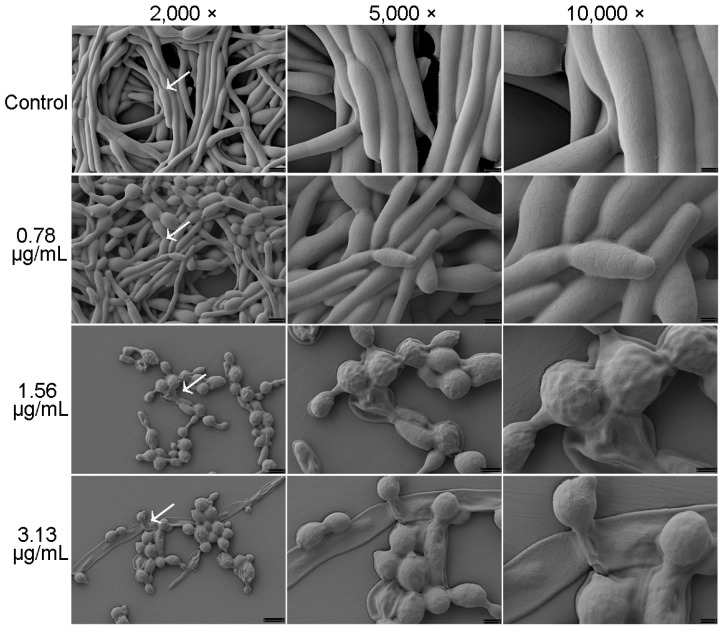
Effect of cocultimycin A against *C. albicans* biofilm observed by SEM. *C. albicans* cells cultured in polystyrene cell culture dish for 24 h to form biofilm in the absence of cocultimycin A (0.78–3.13 μg/mL) or DMSO (0.1%). The biofilm samples were fixed for 24 h with electron microscope fixative, dehydrated in ethanol solutions, and then coated with gold–palladium. The samples were examined using ZEISS-SEM at magnifications of 2000×, 5000× and 10,000×.

The change in morphology of *C. albicans* in mature biofilms following treatment with cocultimycin A was also observed by SEM. As shown in Figure 5, the biofilms in DMSO-treated samples showed dense mycelial crosslinks with intermingling of yeast-like bacteria. However, with the increase in cocultimycin A concentration, the mycelia became loose, and the ratio of hyphae and spores decreased, although yeast-like cells increased significantly. At 1.56 µg/mL and 3.13 µg/mL of cocultimycin A, crisscrossing true hyphae were absent. Moreover, cells treated with cocultimycin A showed obvious shrinkage and breakage.

### 2.5. Cocultimycin A Influenced the Cell Wall Components of C. albicans

**Figure 6:**
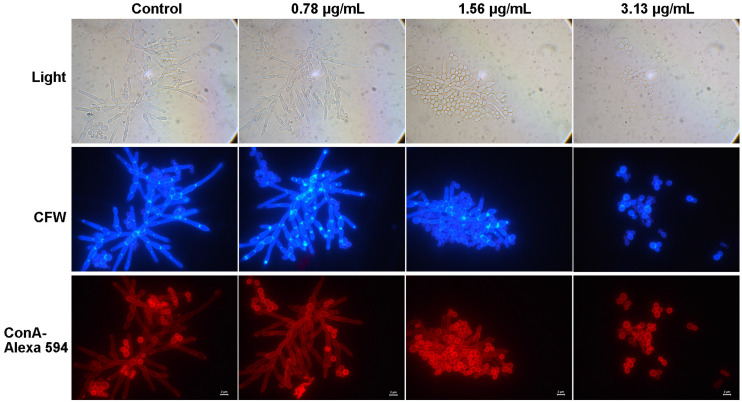
The effect of cocultimycin A on the polysaccharide of the cell wall. The working suspension in 6-well cell culture plate were treated with cocultimycin A (0.78–3.13 μg/mL) or DMSO (0.1%) for 24 h. Biofilm cells were washed with PBS buffer 3 times and stained with CFW or ConA–Alexa 594. The samples were observed by fluorescence microscope at a magnification of 100×, scale bar is 2 μm.

The cell wall plays an important role in the maintenance of cell morphology, host recognition, and immune recognition and is closely related to adhesion, hyphal formation, and crosslinking. Chitin, glucan, and mannan are important components of the cell wall. The adhesion and mycelial formation of *C. albicans* were significantly inhibited by cocultimycin A, which led us to study the effect of cocultimycin A on the cell wall of biofilms by staining these components. CFW was used to stain chitin and glucan, as it can bind to these polysaccharides at the β-junctions. ConA is a protein that binds to mannan and can be used to label mannan and related glycoproteins using fluorescently labeled proteins. As shown in Figure 6, for both CFW and ConA staining, no significant change was observed between the cells treated with cocultimycin A or DMSO.

### 2.6. Cocultimycin A Affected Gene Expression Related to C. albicans Biofilm Formation

**Figure 7:**
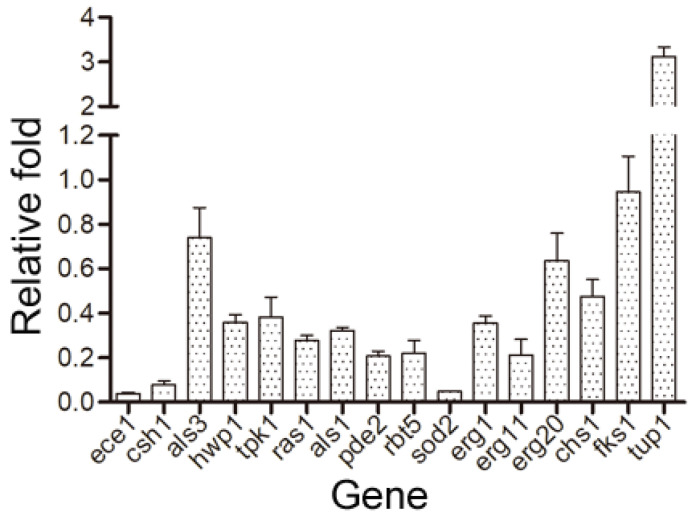
Changes of gene expression in biofilm cells treated with cocultimycin A. The working suspension incubated in a 6-well culture plate was treated with cocultimycin A (1.56 μg/mL) or DMSO (0.1%) for 12 h at 37 °C. The expression of the target genes was detected using QRT-PCR. Relative changes of target genes expression were determined by the 2^−ΔΔC^_T_ method. The results were means ± SEM for three independent experiments.

Several regulatory genes are associated with biofilm formation and are involved in yeast adhesion, hyphal formation, and virulence. In this study, the expression of these genes associated with biofilm formation following treatment with cocultimycin A was evaluated using qRT-PCR, and the functional description of the gene-encoded proteins was obtained from the CGD database (http://www.candidagenome.org/; accessed on 18 February 2021). Compared with the control group, the expression of several genes significantly changed in biofilm cells treated with 1.56 µg/mL cocultimycin A (Table 1). Compared with the control group, biofilms treated with cocultimycin A showed significant down-regulation in terms of the expression of genes related to adhesion and hyphal formation, such as *ece1*, *ras1*, *csh1*, *als1*, *tpk1*, *pde2*, *rbt5*, *als3*, and *hwp1*, especially *ece1* and *csh1*, which were down-regulated 10-fold. In contrast, the expression of the negative regulatory factor *tup1* in biofilm formation was significantly up-regulated (3-fold). Meanwhile, the expression of regulatory genes related to plasma membrane integrity, rigidity, and mobility, such as *sod2*, *erg1*, *erg11*, and *erg20* was significantly down-regulated, with *sod2* being down-regulated over 10-fold. Among genes related to extracellular polysaccharide synthesis in the biofilm matrix and the cell wall, such as *chs1* and *fks1*, only the chitin synthesis-related gene *chs1* was down-regulated two-fold (Figure 7).

### 2.7. Inhibitory Activity of Cocultimycin A against Planktonic C. albicans

Although the activity of cocultimycin A has been reported, the culture medium, pH value, and inoculation dose were not the same as in this study [27]. In order to explore whether the anti-biofilm activity of cocultimycin A is related to its inhibitory activity against planktonic cells, we also re-evaluated the inhibitory activity of cocultimycin A against planktonic *C. albicans* under biofilm culture conditions. Amphotericin B and caspofungin were used as positive controls, and their MICs and MFCs are listed in Table 2. The MICs of cocultimycin A, amphotericin B, and caspofungin were 1.56 µg/mL, 0.39 µg/mL, and 0.1 µg/mL, respectively, while the MFCs were 6.25 µg/mL, 0.78 µg/mL, and 0.4 µg/mL, respectively.

## 3. Materials and Methods

### 3.1. Fungal Strains and Growth Conditions

*C. albicans* strain ATCC10231 was cultured in a yeast extract peptone dextrose (YEPD) medium (1% yeast extract, 2% peptone, and 2% D-glucose) at 37 °C until logarithmic growth was achieved [35]. The cells were then harvested through the use of centrifugation, washed with phosphate-buffered saline (PBS), and diluted to 1 × 10^6^ cells/mL with RPMI-1640 medium (pH = 6.2, Invitrogen, Lofer, Austria) for use as the working suspension for biofilm formation experiments. For biofilm formation, the working suspension was incubated at 37 °C without shaking.

### 3.2. Effects on Biofilm

The biofilms’ total biomass was analyzed using the crystal violet staining methodology [36]. The working suspension was seeded into a 96-well polystyrene cell culture plate (100 μL/well) (Corning Incorporated, New York, NY, USA) and treated with the compounds (cocultimycin A, amphotericin B, and caspofungin), with the final concentration ranging from 0.78 to 6.25 µg/mL at 37 °C for 2 h, 12 h, 24 h and 48 h. A 0.1% DMSO-treated sample was used as the control. The solution was discarded, and each well was lightly washed with PBS (pH = 6.5) twice to remove the non-adhesive cells. Biofilms were first fixed with methanol (100 μL/well) for 15 min and then dried at room temperature. In this experiment, 100 μL of 0.1% crystal violet solution was then added to each well for staining at room temperature for 30 min, and the excess was removed by washing the biofilms three times with PBS. Finally, 100 μL of 100% ethanol was added to each well, the wells were then incubated at room temperature for 1 h, and the absorbance was measured at 595 nm using a multi-label reader (EnVision2014, PerkinElmer, Hopkinton, MA, USA). The wells with RPMI-1640 medium served as blank controls. The rate of biofilm formation (%) was calculated using the equation (A_595 drug_ − A_595 blank_)/(A_595 DMSO_ − A_595 blank_) × 100%.

To determine the disruption activity of compounds on mature biofilms, the working suspension was seeded in a 96-well plate (100 μL/well) and incubated at 37 °C for 24 h. After removing the planktonic fungi with PBS, compounds (from 0.78 to 25 μg/mL) or 0.1% DMSO were then added and treated with another 24 h at 37 °C. The next steps were the same as the above inhibition experiment.

### 3.3. XTT Reduction Assay

The inhibitory ability of cocultimycin A on the biofilm metabolic activity was detected by using the XTT (2,3-bis-(2-methoxy-4-nitro-sulfophenyl)–2H-tetrazolium-5-carboxanilide, Sigma, St. Louis, MO, USA) reduction assay as previously described with minor modifications [37,38,39]. The formation of mature biofilms and the treatment of compounds were conducted as in the crystal violet staining method. The non-adhesive cells were removed by gently washing with PBS thrice, and then the XTT working solution was added and incubated at 37 °C for 2 h in the dark. Absorbance was measured at 490 nm using a multi-label reader (EnVision2014, PerkinElmer, USA). The wells without working suspension served as blank controls. The experiment was repeated thrice with three replicates for each experiment. The relative metabolic activity (%) was calculated using the equation (A_490 drug_ − A_490 blank_)/(A_490 DMSO_ − A_490 blank_) × 100%.

### 3.4. Crystal Violet Staining

The working suspension (2 mL/well) was dded to a 6-well polystyrene cell culture plate (Corning Incorporated, NY, USA) and treated with various concentrations of cocultimycin A (0.78–6.25 µg/mL) or 0.1% DMSO at 37 °C for 12 h, 24 h or 48 h. The plate was washed gently with PBS thrice and inverted for drying. In this experiment, 1 mL of crystal violet solution (0.1%, Sigma) was added to each well for staining at room temperature in the dark for 20 min. The plate was then washed with PBS thrice and observed under a light microscope at 10× magnification (DM2500 inverted, Leica, Wetzlar, Germany).

### 3.5. Cell Surface Hydrophobicity Assay

Cell surface hydrophobicity (CSH) was determined through the use of the water–hydrocarbon two-phase method using methylbenzene as the organic phase, and the assay was performed as previously described with minor modifications [40]. Briefly, the working suspension in 6-well polystyrene cell culture plates (2 mL/well) was treated with cocultimycin A (0.78, 1.56 and 3.13 µg/mL) or 0.1% DMSO for 12 h at 37 °C. The biofilm cells were resuspended in 0.8 mL PBS buffer, and the OD_600_ was detected as A0. Next, 0.2 mL of methylbenzene (Sigma) was added to the suspension, vortexed for 3 min, and settled for 10 min for phase separation. The lower aqueous phase was collected, and OD_600_ was measured as A1. The experiment was repeated thrice with three replicates for each experiment. The hydrophobicity was calculated using the equation (1 − A1/A0) × 100%.

### 3.6. Human Oral Epithelial Cells (HOEC) Adhesion Assay

The HOEC cells that were grown in a cell culture dish with Dulbecco’s modified eagle medium (DMEM) containing 10% fetal bovine serum (FBS) were digested with trypsin (Solarbio, Bejing, China) and diluted in RPMI-1640 medium to 1 × 10^5^ cells/mL. *C. albicans* cells were diluted to 1 × 10^5^ cells/mL in YEPD broth and treated with cocultimycin A (0.39–3.13 µg/mL) or 0.1% DMSO for 12 h at 30 °C. Then, the *C. albicans* cells were harvested via centrifugation at 5000 revolutions per minute (rpm) for 5 min and resuspended in RPMI-1640 to a final concentration of 1 × 10^7^ cells/mL. Then, 0.1 mL of *C. albicans* culture was mixed with 0.1 mL of HOEC culture and co-incubated at 37 °C for 1 h. After incubation, the adhesion of *C. albicans* cells to HOEC cells was observed under a light microscope at 40× magnification and photographed for recording (DM2500 inverted, Leica, Wetzlar, Germany). The number of *C. albicans* cells adhering to 100 randomly selected HOEC cells was determined. The experiment was repeated thrice with three replicates for each experiment. The relative adhesion rate (%) was calculated using the equation (Number _cocultimycin A_/Number _DMSO_) × 100%.

### 3.7. Detection of Yeast-to-Hypha Phase Transition

The working suspension was incubated in a 96-well polystyrene cell culture plate (100 μL/well) for 2 h at 37 °C, and non-adherent cells were removed by washing with PBS. Serially diluted cocultimycin A (0.39–3.13 µg/mL) in RPMI-1640 medium was added and incubated at 37 °C. The cell morphology was observed at 3, 6, and 9 h using a light microscope under 40× magnification (DM2500 inverted, Leica). Samples treated with 0.1% DMSO were used as controls.

### 3.8. Colony Morphology

Colony morphology was studied according to previously reported methods [41,42]. A drop (0.5 µL) of the working suspension was transferred to a YEPD agar plate containing cocultimycin A (1.56–6.25 µg/mL) or 0.1% DMSO. After incubation at 37 °C for 7 days, the hyphae of the colonies were observed under a light microscope at 10× magnification (DM2500 inverted, Leica).

### 3.9. Scanning Electron Microscopy (SEM)

In this study, 4 mL of the working suspension was added to 35 mm cell culture plates (Corning Incorporated, NY, USA) and treated with cocultimycin A (0.78–3.13 µg/mL) or 0.1% DMSO for 24 h at 37 °C. The biofilm samples for SEM analysis were prepared according to a previously reported protocol with minor modifications [43]. Briefly, the biofilm was gently washed with PBS, immersed in 2.5% glutaraldehyde at 4 °C overnight, and washed again with PBS. The biofilm was then dehydrated by using a series of different concentrations of ethanol (30%, 50%, 70%, 80%, 85%, 90%, and 95%), immersed for 10 min in 100% ethanol, and then dried in a desiccator. After sputter-coating with gold–palladium, the samples were analyzed using a ZEISS-SEM (Crossbeam340, Hitachi, Tokyo, Japan) at 2000×, 5000×, and 10,000×.

### 3.10. Cell Wall Staining Assay

In this experiment, 2 mL of the working suspension was added to 6-well polystyrene cell culture plates and treated with cocultimycin A (0.78–3.13 µg/mL) or 0.1% DMSO for 24 h at 37 °C. The biofilm was washed gently with PBS thrice. After adding 4% (*w*/*v*) paraformaldehyde (PFA) for 30 min at ambient temperature, the biofilm was again washed with PBS thrice, and then the staining solution was added. For concanavalin A (ConA) staining, biofilms were incubated with ConA–Alexa594 (Invitrogen Molecular Probes, Carlsbad, CA, USA) at 37 °C for 15 min, and calcofluor white (CFW) staining was performed with 100 µg/mL Fluorescent Brightener 28 [Sigma Aldrich (Shanghai) Trading Co., Ltd., Shanghai, China] in PBS for 3 min. Then, the samples were observed under a fluorescence microscope at a magnification of 100× (DM2500 inverted, Leica).

### 3.11. Quantitative Real-Time Polymerase Chain Reaction (qRT-PCR) Assay

The qRT-PCR assay was conducted as described previously [28]. In this experiment, 10 mL of the working suspension was incubated in a 90 mm polystyrene cell culture plate and treated with cocultimycin A (1.56 µg/mL) or 0.1% DMSO for 12 h at 37 °C. The total RNA of the biofilm was extracted and purified using a rapid fungal RNA extraction kit (Beijing Coolaber Technology Co., Ltd., Beijing, China). The overall quality of the RNA was analyzed via agarose gel electrophoresis, following which cDNA was obtained through a reverse transcription reaction using a reverse transcription kit (RR037A; TaKaRa Biotechnology, Osaka, Japan. SYBR^®^ Green qPCR SuperMix (TransGen Biotech, Beijing, China) was used for qRT-PCR analysis. The primers used are listed in Table S1. The expression of each gene was normalized to that of *β-actin*, and the relative expression of each target gene was calibrated against the corresponding expression in DMSO-treated *C. albicans*, which served as the control. The C_T_ values provided by the FTC-3000 Real-Time PCR Detection System (Funglyn Biotech Inc., Toronto, Canada) are easily imported into Microsoft Excel. Experiments were conducted in independent triplicates to generate a mean value. Relative changes in gene expression were determined using the 2^–ΔΔC^_T_ method as previously described [44]. ΔΔC_T_ was calculated using the following equation:

ΔC_T_ (_test sample_) = C_T_ (_cocultimycin A target gene_) − C_T_ (_cocultimycin A_
*_β-actin_*), ΔC_T_ (_calibration sample_) = C_T_ (_DMSO target gene_) −  C_T_ (_DMSO_
*_β-actin_*), ΔΔC_T_ = Δ C_T_ (_test sample_) − ΔC_T_ (_calibration sample_)

### 3.12. Detection of the Inhibitory Activity of Cocultimycin A against Planktonic C. albicans Cells

The minimum inhibitory concentration (MIC) and minimum fungicidal concentration (MFC) of cocultimycin A against *C. albicans* were determined using the double-dilution method based on the Clinical and Laboratory Standards Institute (CLSI) protocol with minor modifications [45]. Briefly, the working suspension was incubated in a 96-well bacterial culture plate (100 μL/well) [Sangon Biotech (Shanghai) Co., Ltd.], after which compounds (cocultimycin A, amphotericin B, and caspofungin) were added at a final concentration ranging from 0.025 to 25 µg/mL. The sample treated with 0.1% DMSO was used as the control. The plate was incubated at 37 °C for 24 h, and the MIC was defined as the lowest concentration of cocultimycin A that showed no growth of *C. albicans*. To determine the MFC, 100 µL cultures in the wells containing cocultimycin A below the MIC were inoculated into YEPD agar plates and incubated at 37 °C for 24 h. The MFC was the lowest concentration of cocultimycin A that showed fewer than 3 colonies on the YEPD agar plates [46].

### 3.13. Statistical Analysis

GraphPad Prism version 8.0 (GraphPad Software, Inc., San Diego, CA, USA) was used for all statistical analyses. Statistical comparisons were performed using one-way ANOVA. Statistical significance was considered at a *p* value of <0.05. Data are represented as means and standard error of the mean (SEM) for three separate biological replicates, including three technical replicates each, unless otherwise specified.

## 4. Discussion

The correlation between the mortality from invasive candidiasis and biofilm formation has been elucidated with strong evidence [47,48]. As the most common cause of invasive candidiasis, *C. albicans* forms biofilms more easily than other *Candida* species, such as *C. tropicalis* and *C. glabrata*. The biofilms of *C. albicans* are dynamic and highly structured three-dimensional networks composed of a large number of hyphae and ECM, which are resistant to antifungal drugs, particularly azoles [49,50,51]. Therefore, for antifungal drugs, especially *C. albicans*, besides the inhibition of planktonic cells, inhibition of biofilms should be another important indicator to evaluate their antifungal activity. In previous studies, cocultimycin A, an antifungal antibiotic with a novel skeleton, showed good in vitro inhibitory activity against planktonic fungal cells. In this study, we first conducted an in-depth evaluation of its impact on the biofilm morphology of *C. albicans*. Cocultimycin A showed good long-term (from 12 h to 48 h) inhibitory activity on the formation of biofilms and disrupted mature biofilm at high concentration (25 μg/mL) but reduced the metabolic activity of mature biofilms at lower concentrations. Meanwhile, the transition from yeast to hypha phase, adhesion ability and CSH related to biofilms could all be inhibited by cocultimycin A.

In this study, cocultimycin A also showed good inhibitory activity against planktonic *C. albicans*, with MIC of 1.56 µg/mL and MFC of 6.25 µg/mL, which is consistent with the effective inhibitory concentration of cocultimycin A on biofilm formation. In addition, for mature biofilms, cocultimycin A can only reduce the biomass of biofilm to 68.94% at 25 μg/mL. However, 6.25 µg/mL cocultimycin A reduced the metabolic activity of biofilm to 8.96%, while biomass only down-regulated to 89.55%. So, the effect of cocultimycin A on the vitality of *C. albicans* cells should be the main mechanism for its biofilm formation inhibitory activity, especially the clearance of mature biofilms.

At present, the mechanism of cocultimycin A against fungi has not been reported. In this study, the biofilm cells treated with cocultimycin A also showed shrinkage and cracks under SEM, which indicated that cell barriers, such as cell walls or membranes, may be damaged. Together, several genes related to cell membrane synthesis and homeostasis, such as *sod2*, *erg1*, *erg11* and *erg20*, were down-regulated in the biofilm treated with cocultimycin A. Among them, the expression of *sod2* downregulated by more than 10 times. *Sod2* encodes superoxide dismutase, which is a metal-containing antioxidant enzyme that reduces harmful free oxygen radicals produced during normal metabolic cellular processes to oxygen and hydrogen peroxide [52,53]. *Erg1* encodes squalene epoxidase, which plays a key role in the synthesis of ergosterol, an essential component of the cell membrane [54]. Moreover, *erg11* and *erg20* were also associated with the synthesis of ergosterol [55]. So, all these results suggested that the antifungal activity of cocultimycin A, including biofilm and planktonic cells, may be related to the cell membrane homeostasis regulation pathway. Cell membrane damage and lipid regulation pathways should be key targets for studying the mechanism of cocultimycin A.

On the other hand, in the case of biofilms treated with cocultimycin A, the adhesion and mycelial formation ability, CSH and related regulatory genes were also significantly down-regulated, indicating that adhesion and mycelial formation are related to the inhibitory activity of cocultimycin A on biofilm formation. However, whether cocultimycin A directly inhibits the expression of these genes or indirectly down-regulates their expression by reducing cell metabolic activity still requires further research. In addition, the down-regulation of *csh1* and *ece1* was the most significant, exceeding 10 times. Compared with other genes, *csh1* and *ece1* perhaps play a more critical role in the biofilm formation inhibitory activity of cocultimycin A.

Among the current antifungal agents used clinically to treat invasive infections, amphotericin B is highly toxic, and azole drugs have weak bactericidal activity on biofilms and serious drug resistance, and echinocandins have a short half-life and low oral bioavailability, limiting their clinical application. However, in contrast to the rising rate of fungal infections and the increase in drug-resistant fungi, the development of antifungal drugs is relatively slow. Therefore, the discovery of new antifungal drugs is critical. Cocultimycin A showed remarkable inhibitory activity against both planktonic and biofilm of *C. albicans* with relatively low cytotoxicity. Mature biofilms can resist the inhibition of cocultimycin A, but at bactericidal concentrations, the viability of cells decreased significantly, which indicated that the invasion ability of *C. albicans* should be significantly reduced. In summary, cocultimycin A could be a potential leading antifungal compound, although its activity needs further verification in vivo, and its mechanism needs further clarification.

## Figures and Tables

**Table 1:** Relative multiples of target gene expression in cocultimycin A-treated biofilm compared to DMSO-treated sample.

Gene	Mean	SEM	Gene Description
*ece1*	0.038	0.006	Candidalysin, cytolytic peptide toxin essential for mucosal infection; hypha-specific protein; regulated by Rfg1, Nrg1, Tup1, Cph1, Efg1, Hog1, farnesol, phagocytosis; fluconazole-induced; rat catheter and Spider biofilm induced
*ras1*	0.277	0.023	RAS signal transduction GTPase; regulates cAMP and MAP kinase pathways; role in hyphal induction, virulence, apoptosis, heat-shock sensitivity; nonessential; plasma membrane-localized; complements viability of *S. cerevisiae ras1 ras2* mutant
*sod2*	0.048	0.002	Mitochondrial Mn-containing superoxide dismutase; protection against oxidative stress; homotetramer active; N-terminal 34 amino acids removed on mitochondrial import; H_2_O_2_-induced -, alkaline-downregulated, farnesol-induced
*erg1*	0.355	0.032	Squalene epoxidase, epoxidation of squalene to 2,3(S)-oxidosqualene; ergosterol biosynthesis; allylamine antifungal drug target; NADH-reducing cofactor but *S. cerevisiae Erg1* uses NADPH; flow model biofilm induced; Spider biofilm repressed
*erg11*	0.212	0.070	Lanosterol 14-alpha-demethylase; cytochrome P450 family; role in ergosterol biosynthesis; target of azole antifungals; may contribute to drug resistance; azole or flow model biofilm induced; drug treated biofilm induced; hypoxia-regulated
*csh1*	0.078	0.016	Aldo-keto reductase; role in fibronectin adhesion, cell surface hydrophobicity; regulated by temperature, growth phase, benomyl, macrophage interaction; azole resistance associated; Spider biofilm induced; rat catheter biofilm repressed
*als1*	0.323	0.000	Cell-surface adhesin; adhesion, virulence, immunoprotective roles; band at hyphal base; *Rfg1*, *Ssk1*, Spider biofilm induced; flow model biofilm repressed; CAI-4 strain background effects; promoter bound *Bcr1*, *Tec1*, *Efg1*, *Ndt80*, and *Brg1*
*erg20*	0.636	0.123	Putative farnesyl pyrophosphate synthetase involved in isoprenoid and sterol biosynthesis, based on similarity to *S. cerevisiae Erg20p*; likely to be essential for growth, based on an insertional mutagenesis strategy
*tpk1*	0.383	0.089	cAMP-dependent protein kinase catalytic subunit; *Tpk2* isoform; control of morphogenesis and stress response; WT nuclear localization requires *Bcy1*; produced during stationary, not exponential growth; rat catheter and Spider biofilm induced
*chs1*	0.475	0.077	Chitin synthase; essential; for primary septum synthesis in yeast and hyphae; one of several chitin synthases; enzymatically activated by proteolytic processing; complements defects of *S. cerevisiae chs1* or *chs2*; Spider biofilm repressed
*rbt5*	0.220	0.058	GPI-linked cell wall protein; hemoglobin utilization; *Rfg1*, *Rim101*, *Tbf1*, Fe regulated; *Sfu1*, *Hog1*, *Tup1*, serum, alkaline pH, antifungal drugs, geldamycin repressed; *Hap43* induced; required for RPMI biofilms; Spider biofilm induced
*fks1*	0.945	0.016	Catalytic subunit of 1,3-beta-D-glucan synthase; involved in cell wall synthesis and maintenance; localizes to sites of cell wall remodeling; *FKS1* has a paralog, *GSC2*, which arose from the whole-genome duplication
*tup1*	3.117	0.022	Transcriptional corepressor; represses filamentous growth; regulates switching; role in germ tube induction, farnesol response; in repression pathways with *Nrg1*, *Rfg1*; farnesol upregulated in biofilm; rat catheter, Spider biofilm repressed
*pde2*	0.207	0.022	High-affinity cyclic nucleotide phosphodiesterase; moderates signaling by cAMP; required for virulence, switching, cell wall, hyphal, not pseudohyphal growth; expressed shortly after hyphal induction; rat catheter and Spider biofilm induced
*als3*	0.739	0.136	Cell wall adhesin; epithelial adhesion, endothelial invasion; alleles vary in adhesiveness; immunoprotective in mice; binds SspB adhesin of *S. gordonii* in mixed biofilm; induced in/required for Spider biofilm; flow model biofilm repressed
*hwp1*	0.359	0.034	Hyphal cell wall protein; host transglutaminase substrate; opaque-, a-specific, alpha-factor induced; at MTLa side of conjugation tube; virulence complicated by URA3 effects; Bcr1-repressed in RPMI a/a biofilms; Spider biofilm induced

**Table 2:** Inhibitory activity of compounds against planktonic *C. albicans*.

Drug	MIC (μg/mL)	MFC (μg/mL)
Cocultimycin A	1.56	6.25
Amphotericin B	0.39	0.78
Caspofungin	0.10	0.40

## Data Availability

Data are contained within the article and Supplementary Materials.

## Supplementary Materials

The following supporting information can be downloaded at: https://www.mdpi.com/article/10.3390/ijms242317026/s1.
