# Supplementary material for: Anti-Biofilm Activity of Cocultimycin A against Candida albicans
Source: Int J Mol Sci. 2023 Dec 1;24(23):17026. doi: 10.3390/ijms242317026 (PMC10707031; doi:10.3390/ijms242317026)
Supplement: Supplementary file 1 [file ijms-24-17026-s001.zip › ijms-2680059-supplementary.pdf]

Table S1 Primers of genes used for RT-PCR

Gene	Primer-forward	Primer-reverse
ece1	GCTGGTATCATTGCTGATAT	TTCGATGGATTGTTGAACAC
ras1	GTGGTGGTGTTGGTAAATC	TTCTTGTCCAGCAGTATCT
sod2	AAACTTGGCTCCTGTCTC	GTATCACCATTGGCTTTG
erg1	CTCCTTCTGCTGCTAACG	CCAACTGTCATACCACCC
erg11	TTGGTGGTGGTAGACATAG	CTGCTGGTTCAGTAGGT
csh1	CTCAATCCT TTACCGTTC	AGAGTTGTTGGGTTTATT C
als1	ATTGGTAAAGTAACTGTACCA	CACATTGAATATGCCATGTG
erg20	TTACCCGTGGCATTAGCAATGTA	TCCCAAGGGAATCAAAATGTCTC
tpk1	CAAAGCAGCCATACATCC	AGAAGTTCAAGATGTGACTTAT
chs1	CTGACAAGAGCCAACACTGC	CGCCTCTTGATGGTGATGAT
rbt5	CTGCTAAAGAAACCACTGCTG	GCTTCAACGGAAACAGAAGC
fks1	CGTGAAATTGATCATGCCTGTAC	AACCCTTCTGGGCTCCAAA
tup1	CTTGGAGTTGGCCCATAGAA	TGGTGCCACAATCTGTTGTT
β-actin	ACCGAAGCTCCAATGAATCC	CCGGTGGTTCTACCAGAAGAG
pde2	TGCTGTGGGACATTGGAG	GGCGGAAATTATGGAACG
als3	TAATGCTGCTACGTATAATT	CCTGAAATTGACATGTAGCA
hwp1	TGGTGCTATTACTATTCCGG	CAATAATAGCAGCACCGAAG
